# Antimicrobial Activity of Lipophilic Gallium-Based Compounds: An Emerging Frontier in Metal-Based Antimicrobials

**DOI:** 10.3390/antibiotics15100975

**Published:** 2026-10-02

**Authors:** Nalin Abeydeera, Bishnu Pant, Guanyu Chen

**Affiliations:** 1 Department of Medicine, University of California San Francisco, San Francisco, CA 94143, USA; 2 Department of Inflammation and Immunity, Cleveland Clinic Lerner Research Institute, Clevland, OH 44195, USA; pantb@ccf.org; 3 Department of Chemistry and Biochemistry, Kent State University, Kent, OH 44242, USA; gchen10@kent.edu

**Keywords:** antimicrobial resistance, Ga-based antimicrobials, lipophilicity-driven design

## Abstract

Antimicrobial resistance (AMR) is an increasing global health threat that underscores the urgent need for antimicrobial agents with mechanisms of action distinct from those of conventional antibiotics. Gallium (Ga) has emerged as a promising metal-based antimicrobial owing to the chemical similarity of Ga(III) to Fe(III), which enables it to interfere with bacterial iron acquisition and utilization. In contrast to Fe(III), Ga(III) is redox-inactive under physiological conditions and cannot support the redox reactions required for many iron-dependent cellular processes, thereby disrupting bacterial metabolism. This review highlights recent advances in gallium-based antimicrobial strategies, with particular emphasis on the role of ligand coordination in modulating the antibacterial activity of Ga(III) against drug-resistant pathogens. Emerging evidence indicates that coordination with lipophilic ligands can enhance the cellular update and accumulation of Ga(III) and may confer additional antibacterial effects beyond Fe(III) mimicry alone. These findings suggest that ligand design is a key strategy for tuning the physicochemical properties and antimicrobial activity of gallium complexes. Rational development of ligand-coordinated Ga(III) complexes may therefore provide new therapeutic approaches for combating multidrug-resistant bacterial infections.

## 1. Introduction

Antimicrobial resistance (AMR) has become a major global public health concern, posing a significant threat to the effective prevention and treatment of infectious diseases [1]. In 2019, AMR was associated with an estimated 4.95 million deaths worldwide, underscoring its profound impact on healthcare systems and patient outcomes [2]. According to the World Health Organization (WHO), drug-resistant infections are responsible for at least 700,000 deaths annually, with resistance to first-line antimicrobial therapies continuing to rise [3].

Among the pathogens contributing to the AMR crisis, *Pseudomonas aeruginosa* (PA) and *Staphylococcus aureus* (SA) are particularly important because of their widespread prevalence and remarkable capacity to acquire multidrug resistance [4,5]. *P. aeruginosa*, a Gram-negative opportunistic pathogen, is capable of surviving in diverse environmental conditions and frequently colonizes burns, surgical wounds, and medical devices [6]. Its intrinsic resistance mechanisms, together with its ability to form persistent biofilms, make infections extremely difficult to eradicate [7,8,9,10]. More than one-third of clinical isolates have been reported to be resistant to three or more classes of antibiotics [11]. *P. aeruginosa* is also a major cause of healthcare-associated infections, particularly among immunocompromised patients and individuals with cystic fibrosis or cancer, where it is associated with severe morbidity and mortality [12,13,14].

Similarly, *Staphylococcus aureus* is a Gram-positive pathogen that causes diverse infections affecting the skin, respiratory tract, bloodstream, bones, and surgical sites [15,16]. It is responsible for approximately 10% of hospital-acquired infections globally and continues to be a major cause of postoperative and device-associated infections. A key virulence characteristic of *S. aureus* is its remarkable ability to acquire iron from the host, an essential nutrient that supports bacterial growth, metabolism, and pathogenicity [17,18,19]. The rapid emergence of multidrug-resistant *S. aureus* (MDRSA), including methicillin-resistant *S. aureus* (MRSA) and vancomycin-resistant *S. aureus* (VRSA), has significantly reduced the effectiveness of current conventional antibiotics and has contributed to increased morbidity, mortality, and healthcare costs [20,21,22].

The increasing prevalence of multidrug-resistant *P. aeruginosa* and multidrug-resistant *S. aureus*, combined with the limited development of new antibiotics, highlights the urgent need for alternative antimicrobial strategies [23,24,25]. Although the use of metals as antimicrobial agents dates back to ancient civilizations, their clinical application declined following the widespread introduction of organic antibiotics during the mid-twentieth century [26]. In recent years, metal-based antimicrobial agents have attracted renewed attention due to their distinctive physicochemical properties, structural diversity, and capacity to target multiple bacterial pathways simultaneously [27,28,29,30,31,32]. Unlike conventional antibiotics that typically inhibit a single cellular process, metallo-antimicrobials often exert multitargeted effects, reducing the likelihood of resistance development [15,16,17,33,34,35].

Among these metallo-antimicrobials, gallium (Ga) has emerged as a promising antimicrobial agent due to its close chemical resemblance to ferric iron (Fe^3+^), such as similar ionic radius, coordination chemistry, oxidation state, and electronegativity [36,37]. These similarities enable gallium to hijack bacterial iron acquisition systems and interfere with iron-dependent metabolic processes. Since both *P. aeruginosa* and *S. aureus* rely on iron for essential cellular functions and the expression of virulence factors, gallium-based compounds have attracted considerable interest as a novel therapeutic strategy for combating antimicrobial-resistant infections [36,37,38].

Previous reviews have primarily emphasized the antimicrobial mechanisms, biological applications, and translational potential of gallium compounds. However, comparatively less attention has been directed toward understanding how ligand coordination influences the antimicrobial activity of Ga(III) complexes. To address this gap, a comprehensive literature search was conducted using PubMed and Google Scholar to identify relevant studies on gallium-based antimicrobial agents, with particular attention to Ga(III) complexes and their activity against antibiotic-resistant bacteria. Search terms included “antimicrobial resistance”, “gallium-based antimicrobials”, “Ga-based compounds”, and “bacterial iron metabolism”. Relevant references were compiled and managed using EndNote X8. Based on the identified literature, this review focuses on recent advances in ligand-guided Ga(III) antimicrobial design, with particular emphasis on ligand lipophilicity, cellular uptake, complementary ligand-derived mechanisms, and emerging structure–activity relationships.

## 2. Iron Acquisition in Bacteria

**Figure 1:**
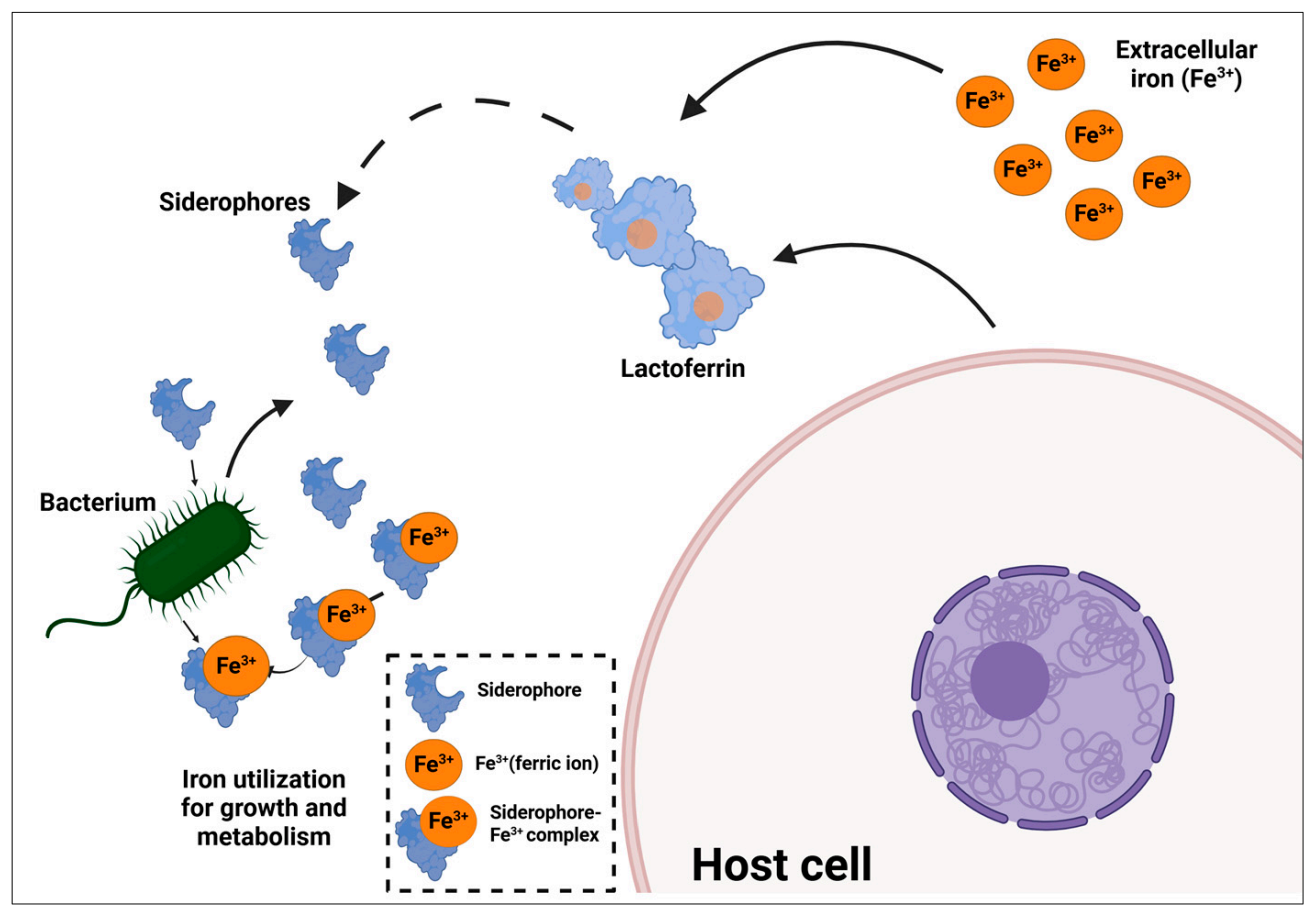
Siderophore-mediated iron acquisition by pathogenic bacteria.

Iron is an essential micronutrient required by nearly all living organisms and functions as a critical cofactor for numerous enzymes involved in vital cellular processes, including DNA synthesis, electron transport, and defense against oxidative stress [39,40]. However, the host tightly controls iron availability as a key component of innate immune defense by sequestering iron in proteins such as transferrin, lactoferrin, ferritin, and hemoglobin, thereby restricting access to this essential nutrient by invading bacteria [41,42,43]. As a result, invading pathogens encounter an iron-limited environment and must employ specialized iron acquisition systems to obtain sufficient iron for survival, growth, and virulence [44]. To overcome the host’s iron-restriction defense, pathogenic bacteria have evolved specialized iron acquisition strategies, the most common of which involves the production of siderophores [45,46] (Figure 1).

Siderophores are low-molecular-weight, high-affinity, iron-chelating molecules that are synthesized and secreted under iron-limited conditions by bacteria [47,48]. Siderophores scavenge ferric iron (Fe^3+^) from host iron-binding proteins by forming highly stable iron–siderophore complexes, which are subsequently recognized and transported into bacterial cells through specific receptor-mediated uptake systems [49,50]. This process enables bacteria to get sufficient iron to support essential metabolic activities, growth, and virulence. More than 500 structurally distinct siderophores have been identified to maximize iron acquisition from diverse host iron sources [51]. Based on their iron-coordinating functional groups, siderophores are generally classified into four major categories: catecholates, hydroxamates, carboxylates, and phenolates, although some possess mixed ligand types that combine features of two or more classes [52,53].

## 3. Gallium as an Iron Mimic: Exploiting the Trojan Horse Strategy Against Bacterial Pathogens

**Figure 2:**
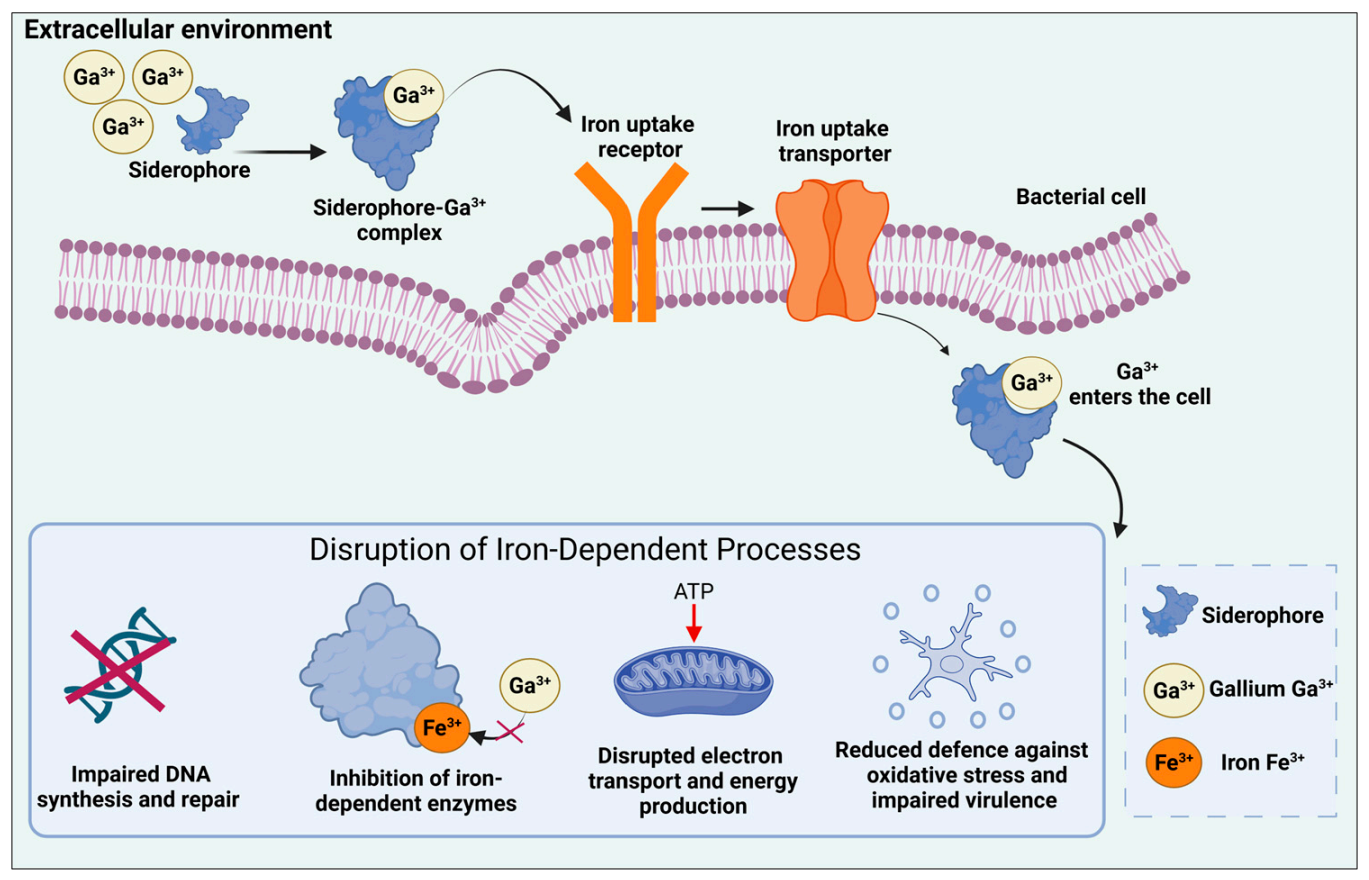
Trojan horse mechanism of gallium (Ga^3+^)-mediated antimicrobial activity.

Gallium (Ga) has emerged as a promising antimicrobial agent because of its close chemical properties to ferric iron (Fe^3+^) [36]. In other words, gallium has a several physicochemical characteristics similar with Fe^3+^, including ionic charge, ionic radius (Ga^3+^ = 0.62 Å vs. Fe^3+^ = 0.65 Å for the high-spin electron configuration), and coordination number (i.e., both ions typically prefer octahedral coordination environments), allowing it to be mistakenly recognized by bacterial iron acquisition systems [54,55,56,57]. This phenomenon, referred to as the “Trojan horse” strategy, enables Ga^3+^ to enter bacterial cells through siderophore-mediated transporters and other iron uptake pathways [58]. However, unlike Fe^3+^, gallium is redox-inactive under physiological conditions and cannot be reduced to Ga^2+^. Consequently, once uptake into bacterial cells, gallium cannot participate in iron-dependent redox reactions that are essential for numerous metabolic processes [55,56,59]. The substitution of Fe^3+^ with Ga^3+^ disrupts the function of iron-containing enzymes involved in DNA synthesis, cellular respiration, oxidative stress defense, and energy production, ultimately inhibiting bacterial growth [60] (Figure 2). Because pathogenic bacteria depend heavily on efficient iron acquisition for survival, biofilm formation, and infection, gallium effectively exploits these iron uptake systems to exert its antimicrobial activity while minimizing the development of resistance [61,62].

## 4. Gallium in Biological Applications

The biomedical application of gallium compounds began in the early 1970s, primarily as radiopharmaceuticals for diagnostic imaging [63]. Radioisotopes of gallium (^67^Ga) have been extensively used to detect malignant tumors, identify sites of infection and inflammation, and evaluate bone-related diseases [62,63]. The success of gallium in diagnostic medicine stimulated further research into its therapeutic potential. Over the past several decades, gallium-based compounds have demonstrated efficacy in treating a variety of conditions, including excessive bone resorption, malignancies, and microbial infections [64,65,66,67]. Among these compounds, gallium nitrate [Ga(NO_3_)_3_], became the first gallium-based drug approved by the U.S. Food and Drug Administration (FDA) [64]. It is administered intravenously for the treatment of cancer-associated hypercalcemia, highlighting the safety and clinical feasibility of gallium-containing therapeutics [68]. Several gallium-based compounds have reached Phase I and Phase II clinical trials. Among these, gallium chloride (GaCl_3_) has been investigated as a treatment for cancer; however, its clinical application has been limited by poor bioavailability following oral and aerosol administration [69]. Oral administration of GaCl_3_ at 400 mg/day resulted in gallium accumulation primarily in the lungs and bones without significant nephrotoxicity, whereas aerosol administration led to the deposition of gallium hydroxide within the respiratory tract [69,70]. These limitations have driven the development of alternative gallium formulations designed to achieve more favorable pharmacokinetic profiles and improved therapeutic efficacy.

## 5. Gallium-Based Antimicrobial Agents

The antimicrobial potential of gallium was first discovered in the early 1930s when Levaditi et al. reported that gallium tartrate effectively treated syphilis in rabbits and *Trypanosoma evansi* infection in mice following intramuscular or intravenous administration [67,71]. Despite these early findings, research on the antimicrobial activity of gallium remained limited for several decades. Researchers became interested again in the early 1970s when showed that Ga^3+^ ions could bind to microbial siderophores and be transported into bacterial cells through iron uptake pathways [72]. Because Ga^3+^ closely resembles Fe^3+^ chemically but cannot be involved in iron-dependent redox reactions, its incorporation into microbial cells disrupts essential iron-dependent metabolic processes, thereby inhibiting microbial growth [73]. This iron-mimetic mechanism laid the foundation for subsequent research into gallium-based antimicrobial agents. However, significant advances in this field were not achieved until the late 1990s, when research on gallium-based compounds expanded substantially [55]. Since then, numerous gallium salts have been evaluated, demonstrating varying degrees of antimicrobial activity against a broad range of clinically relevant pathogens [55,62,74]. Gallium nitrate inhibits the growth of planktonic *P. aeruginosa*, prevents biofilm formation, and disrupts established biofilms, highlighting its potential for treating chronic biofilm-associated infections [75,76]. Other gallium salts, including gallium chloride, have also been reported to inhibit the growth of *P. aeruginosa* and suppress biofilm formation.

## 6. Rational Ligand Design Strategies for Enhancing Ga(III) Antimicrobial Activity

Recent studies have highlighted ligand-mediated gallium delivery as a promising strategy for enhancing the antimicrobial efficacy of Ga(III)-based antimicrobial agents. The Ga^3+^ ion is classified as a hard Lewis acid because of its high charge density and low polarizability and, consequently, exhibits a strong affinity for hard Lewis bases, particularly ligands containing oxygen- (O-) and nitrogen- (N-)-donor atoms [57,72]. Coordination of Ga(III) with lipophilic organic ligands can modulate its physicochemical properties, stability, and cellular uptake, thereby improving its antimicrobial activity [77]. Moreover, if the ligand possesses intrinsic antimicrobial activity, its combination with the iron-mimetic effects of Ga(III) could provide a basis for dual antimicrobial action [78]. This dual-action strategy may therefore provide both improved gallium delivery and complementary antimicrobial effects, potentially enhancing the overall efficacy of Ga(III)-based compounds.

This ligand-mediated effect is supported by gallium citrate, which has demonstrated potent antibacterial activity against Gram-negative bacteria associated with cystic fibrosis, with reported minimum inhibitory concentration (MIC) values below 5 μg/mL [74]. Gallium tartrate has similarly been reported to enhance bacterial gallium uptake, emphasizing the critical role of the coordination environment in determining the biological role of Ga(III). Accordingly, the incorporation of rationally designed lipophilic ligands may offer a strategy to improve bacterial gallium uptake while potentially introducing complementary ligand-derived antimicrobial effects [78].

Gallium maltolate (GaM) represents a notable example of this ligand-guided approach. Maltol (3-hydroxy-2-methyl-4-pyrone) functions as a bidentate O,O-donor ligand with a strong affinity for Ga(III), providing a stable coordination environment while making a neutral Ga(III) complex with properties distinct from those of gallium salts [79]. Its relatively highly lipophilic character facilitates gallium uptake into bacterial cells [16]. Importantly, the antimicrobial activity reported for GaM across different bacterial species demonstrates the potential biological relevance of this ligand-mediated delivery strategy. Gallium maltolate exhibited antibacterial activity against 98 clinical isolates of *Rhodococcus equi*, with MIC values ranging from 8 to 32 μg/mL [80]. Activity has also been reported against *Staphylococcus aureus*, including methicillin-resistant *S. aureus* (MRSA), although higher MIC values of approximately 50–200 μg/mL were observed [81]. Importantly, GaM has also demonstrated promising activity against the Gram-negative pathogen *Pseudomonas aeruginosa*. In murine models of thermal injury followed by *P. aeruginosa* infection, GaM treatment significantly reduced local and systemic bacterial burdens, limited bacterial colonisation, and improved survival. Furthermore, at an equivalent gallium dose of 25 mg/kg, GaM demonstrated greater antibacterial efficacy than gallium nitrate, producing more pronounced inhibition of *P. aeruginosa* growth under comparable experimental conditions. This comparison is particularly relevant to ligand-guided Ga(III) design because the enhanced activity of GaM relative to gallium nitrate suggests that the chemical form in which Ga(III) is delivered can substantially influence its biological performance. Gallium maltolate (GaM) has also demonstrated bactericidal activity against multidrug-resistant strains of *Acinetobacter baumannii*, further highlighting its potential as an antimicrobial agent against clinically challenging Gram-negative pathogens. Taken together with its antimicrobial activity against *R. equi*, *S. aureus* and *P. aeruginosa*, these findings indicate that GaM possesses broad-spectrum antimicrobial activity. More importantly, comparison with gallium nitrate suggests that ligand coordination may enhance the antimicrobial efficacy of Ga(III). Additionally, pyochelin is a siderophore produced by *P. aeruginosa* that normally facilitates bacterial iron acquisition. Complexation of pyochelin with Ga(III) can exploit these bacterial iron-uptake pathways, enhance gallium uptake and result in greater antimicrobial activity against *P. aeruginosa* compared with gallium nitrate (Ga(NO_3_)_3_).

Similarly, deferoxamine (DFO), a strong iron-chelating siderophore, has been investigated as a carrier for targeted delivery of Ga(III) to *P. aeruginosa* [82]. The deferoxamine–gallium complex (DFO–Ga) was designed to exploit the bacterial uptake systems that normally recognize iron-bound DFO, thereby acting as a “Trojan horse” for gallium delivery [83]. DFO–Ga showed antimicrobial activity against both planktonic and biofilm-associated *P. aeruginosa*, with an MIC of 0.032 mM under low-iron conditions. It was also effective against several clinical isolates obtained from cystic fibrosis sputum, eye infections, and wounds. Importantly, DFO–Ga showed enhanced antibacterial activity when combined with gentamicin, producing approximately a six-log reduction in viable cells in mature biofilms. In a rabbit model of *P. aeruginosa* keratitis, treatment with DFO–Ga and gentamicin also reduced corneal infiltrate and final scar size by approximately 50% compared with gentamicin alone [82]. These findings demonstrate the potential of siderophore-mediated Ga(III) delivery and further support the importance of ligand selection in improving the antimicrobial activity of gallium-based compounds.

Bandey et al. developed Galbofloxacin, a rationally designed gallium (III)-siderophore–antibiotic conjugate targeting *Staphylococcus aureus* [84]. This gallium complex consists of a Ga(III)-coordinating desferrichrome siderophore linked to ciprofloxacin through a protease-cleavable serine linker. This design utilizes bacterial iron-acquisition pathways to enhance the uptake of the Ga(III) complex, acting as a “Trojan horse” to deliver both gallium and ciprofloxacin into bacterial cells. Radiolabeled ^67^Ga-Galbofloxacin showed time-dependent and siderophore-mediated bacterial uptake. Also, protease experiments demonstrated cleavage of the serine-containing linker, supporting the proposed mechanism in which the antibiotic is released following bacterial uptake. Importantly, the enhanced antimicrobial activity was not solely due to ciprofloxacin. Galbofloxacin showed an MIC of 93 nM against *S. aureus*, which was much lower than those of free ciprofloxacin (0.9 μM) and the corresponding metal-free (apo) conjugate (0.93 μM). This result indicates that Ga(III) complexation contributes significantly to the improved antibacterial activity. Also, in a mouse model of intramuscular *S. aureus* infection, animals receiving two doses of Galbofloxacin at 9.3 μmol kg^−1^ survived the seven-day study while their bacterial burden progressively decreased. At the same molar dose, ciprofloxacin alone was ineffective, and the infection was fatal within the first day in that treatment group. Overall, these findings demonstrate that rational siderophore-based ligand design can significantly improve the antimicrobial efficacy of Ga(III) complexes by enhancing bacterial uptake and enabling targeted antibiotic delivery. Galbofloxacin therefore represents a promising strategy for developing more potent and selective gallium-based antimicrobial agents.

Another recent example of ligand-guided optimization of Ga(III) antimicrobial complexes was reported by Frank et al., who developed GaSal-2, a water-soluble derivative of the previously described gallium–salophen complex GaSal [85]. GaSal-2 was designed to overcome limitations associated with the aqueous solubility of the parent complex while preserving its ability to target the extracellular hemophore HasAp of *Pseudomonas aeruginosa*. The complex exhibited strong binding to HasAp and inhibited HasAp-mediated activation of the bacterial cell-surface signaling pathway involved in heme acquisition. This targeted interference was accompanied by inhibition of *P. aeruginosa* growth and disruption of established biofilms. Importantly, GaSal-2 showed no detectable toxicity toward human lung fibroblasts or hepatocytes under the conditions examined and demonstrated antipseudomonal activity in a murine lung infection model. Collectively, these findings illustrate how rational modification of the ligand framework surrounding Ga(III) can improve physicochemical properties, such as aqueous solubility, while maintaining biologically relevant target engagement and antimicrobial activity.

Centola et al. demonstrated that coordination of Ga(III) with the salophen ligand produces a dual-action antimicrobial complex capable of disrupting both heme sensing and iron acquisition in *Pseudomonas aeruginosa* [86]. Ga(III)–salophen (GaSal) was shown to interact with the extracellular hemophore HasAp and inhibit activation of the Has heme-sensing pathway, thereby interfering with a signaling system involved in bacterial adaptation to host heme availability. In addition to this extracellular effect, GaSal inhibited bacterial growth through active uptake via a xenosiderophore pathway, although the specific receptor responsible for its internalization was not identified. Following uptake, Ga(III) acts as a redox-inactive Fe(III) mimic, disrupting iron-dependent cellular processes. Thus, the salophen ligand provides more than a coordination scaffold for Ga(III); it contributes to molecular recognition of the Has system while facilitating a distinct route of cellular entry. This dual mechanism highlights how ligand selection can expand the antimicrobial activity of Ga(III) beyond conventional iron mimicry by combining interference with extracellular iron-sensing pathways and intracellular gallium-mediated disruption of iron metabolism.

Further evidence supporting the use of lipophilic ligands in Ga(III)-based antimicrobial design was reported by Wang et al., who developed the lipophilic gallium complex [Ga_2_L_3_(bpy)_2_], containing pharmacophoric ligands L-lawsone (2-hydroxy-1,4-naphthoquinone) and bpy-2,2′-bipyridine [87]. This lipophilic Ga complex showed broad-spectrum antimicrobial activity against both drug-resistant *P. aeruginosa* and methicillin-resistant *S. aureus*, with MIC values of 10 μM (14.8 μg/mL) and 100 μM (148 μg/mL), respectively. Importantly, this activity was observed without the need for iron-limited conditions. In addition, the complex exhibited a slower development of gallium resistance in both *P. aeruginosa* and *S. aureus* following 30 days of repeated exposure, compared with ciprofloxacin. The antimicrobial activity of [Ga_2_L_3_(bpy)_2_] was proposed to arise from a dual mode of action, in which Ga(III) disrupts bacterial iron metabolism while the pharmacophoric ligand; Lawsone contributes to reactive oxygen species (ROS) production. These findings demonstrate that the complexation of lipophilic and biologically active ligands into Ga(III) complexes can provide broad-spectrum antimicrobial activity through complementary mechanisms, supporting the rational design of ligand-guided gallium antimicrobials. The therapeutic potential of gallium-based antimicrobial agents depends not only on their antibacterial efficacy but also on their selectivity toward bacterial cells over mammalian cells. Wang et al. evaluated mammalian cytotoxicity of [Ga_2_L_3_(bpy)_2_] using RAW 264.7 murine macrophage-like cells exposed to concentrations ranging from 10 to 500 μM for 24 h. Remarkably, greater than 90% cell viability was retained even at the highest concentration tested (500 μM), and microscopic examination revealed no apparent changes in cellular morphology. These findings provide evidence that appropriately designed lipophilic Ga(III) complexes can exhibit substantial preferential toxicity toward bacterial cells relative to mammalian cells, supporting the existence of a potentially useful therapeutic window.

**Figure 3:**
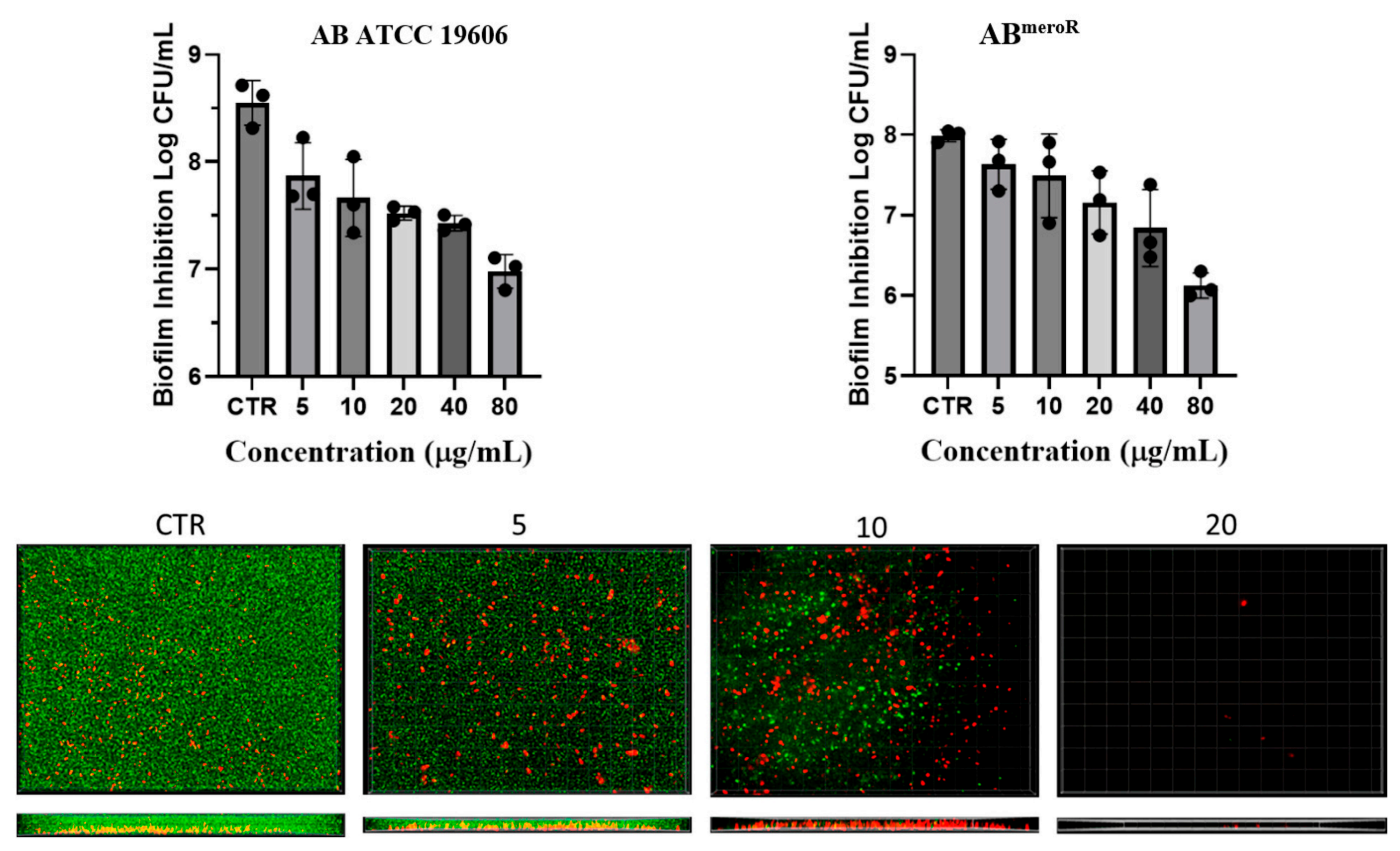
Results of quantification of biofilm inhibition activity of KP46 in Acinetobacter baumannii (**top left**) and Acinetobacter baumannii resistant to meropenem (**top right**). Representative fluorescence images (**bottom panels**) show Live/Dead staining of biofilm. Reproduced from [88], Figure 4, under the Creative Commons Attribution 4.0 International (CC BY 4.0) license.

Also the therapeutic potential of lipophilic Ga(III) complexes has also been investigated through drug repurposing [88]. KP46 [tris(8-quinolinolato)gallium(III)] is an orally bioavailable Ga(III) complex originally developed as an anticancer agent. Importantly, its ligand, 8-hydroxyquinoline (8-hq), is itself a well-known metal-chelating scaffold with reported antimicrobial activity against a range of microorganisms. The antimicrobial properties of 8-hq and its derivatives have been associated with their ability to chelate metal ions and interfere with metal-dependent microbial processes, while their lipophilic character may also facilitate interactions with and penetration of microbial membranes. Therefore, the coordination of Ga(III) with 8-hq is of particular interest, as the ligand may provide additional biological activity rather than serving solely as a carrier for Ga(III). KP46 has recently demonstrated antibacterial activity against *Acinetobacter baumannii*, including carbapenem-resistant *Acinetobacter baumannii* (CRAB) clinical isolates [88]. The complex inhibited planktonic bacterial growth and interfered with early biofilm development (Figure 3).

**Figure 4:**
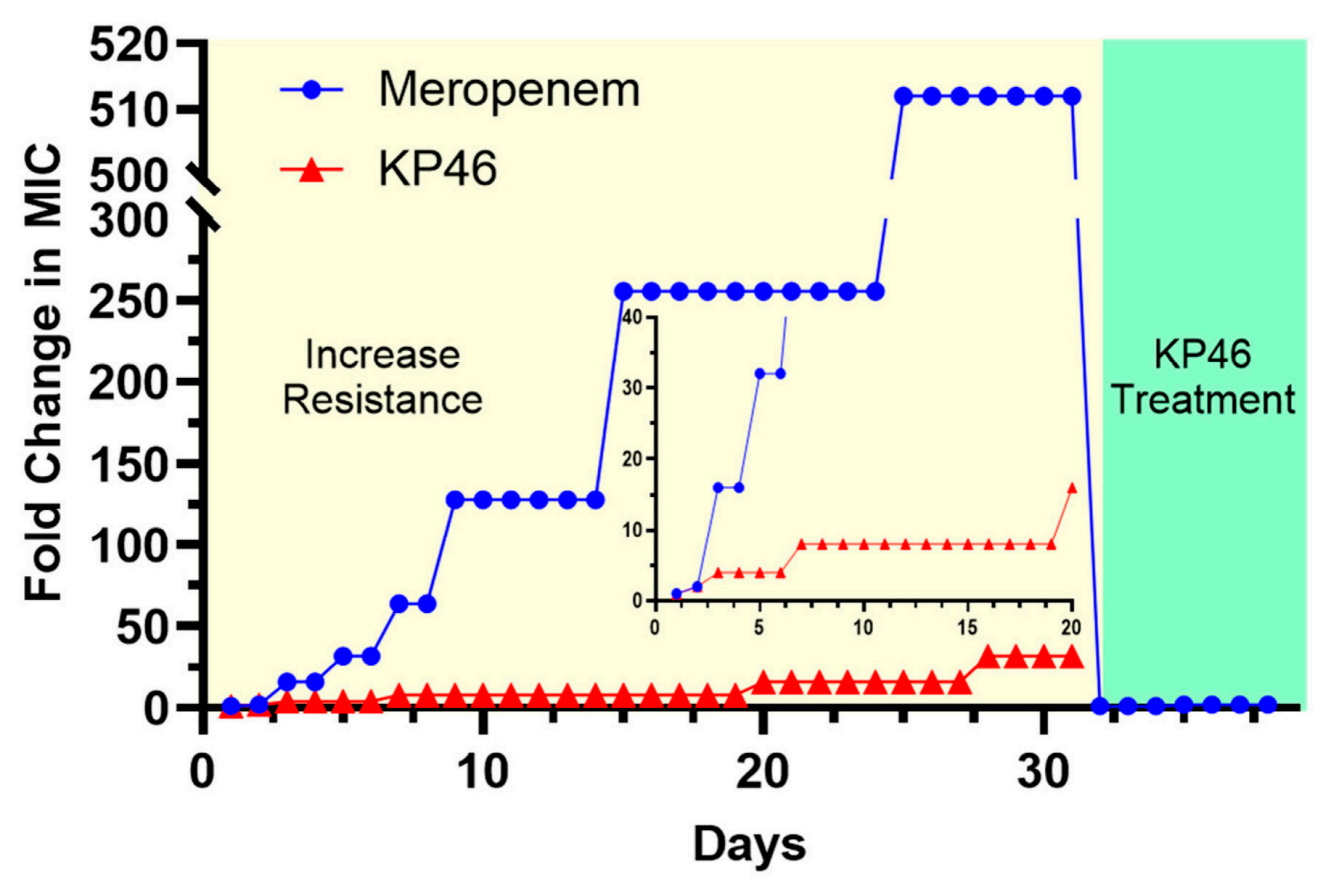
Development of resistance in *A. baumannii* during 31-day serial passaging under sub-inhibitory concentrations of KP46 or meropenem. Reproduced from [88], Figure 5, under the Creative Commons Attribution 4.0 International (CC BY 4.0) license.

Mechanistic studies indicated that KP46 increased intracellular oxidative stress and compromised bacterial membrane integrity, contributing to bacterial cell death. Furthermore, resistance to KP46 developed more slowly than resistance to meropenem, and the complex retained activity against meropenem-resistant isolates (Figure 4).

Together with the established antimicrobial activity of 8-hq, these findings highlight the potential of pairing Ga(III) with biologically active, lipophilic ligands to generate Ga(III) complexes with enhanced and complementary antimicrobial properties. More recently, lipophilicity-guided ligand design has been explored as a strategy to improve the antibacterial and antibiofilm activity of Ga(III) complexes [89]. Chen et al. investigated the influence of ligand structure on antimicrobial activity using six conjugated heterocyclic ligands: maltol (3-hydroxy-4-pyrone), kojic acid (5-hydroxy-2-(hydroxymethyl)-4-pyrone), meconic acid (3-hydroxy-4-oxo-4H-pyran-2,6-dicarboxylic acid), hinokitiol (4-isopropyl-2-hydroxycyclohepta-2,4,6-trien-1-one), tropolone (2-hydroxycyclohepta-2,4,6-trien-1-one), and phthiocol (2-hydroxy-3-methyl-1,4-naphthoquinone). The antimicrobial activities of these ligands and their corresponding Ga(III) complexes were evaluated against both Gram-positive and Gram-negative bacteria, including *S. aureus* and *P. aeruginosa*.

**Figure 5:**
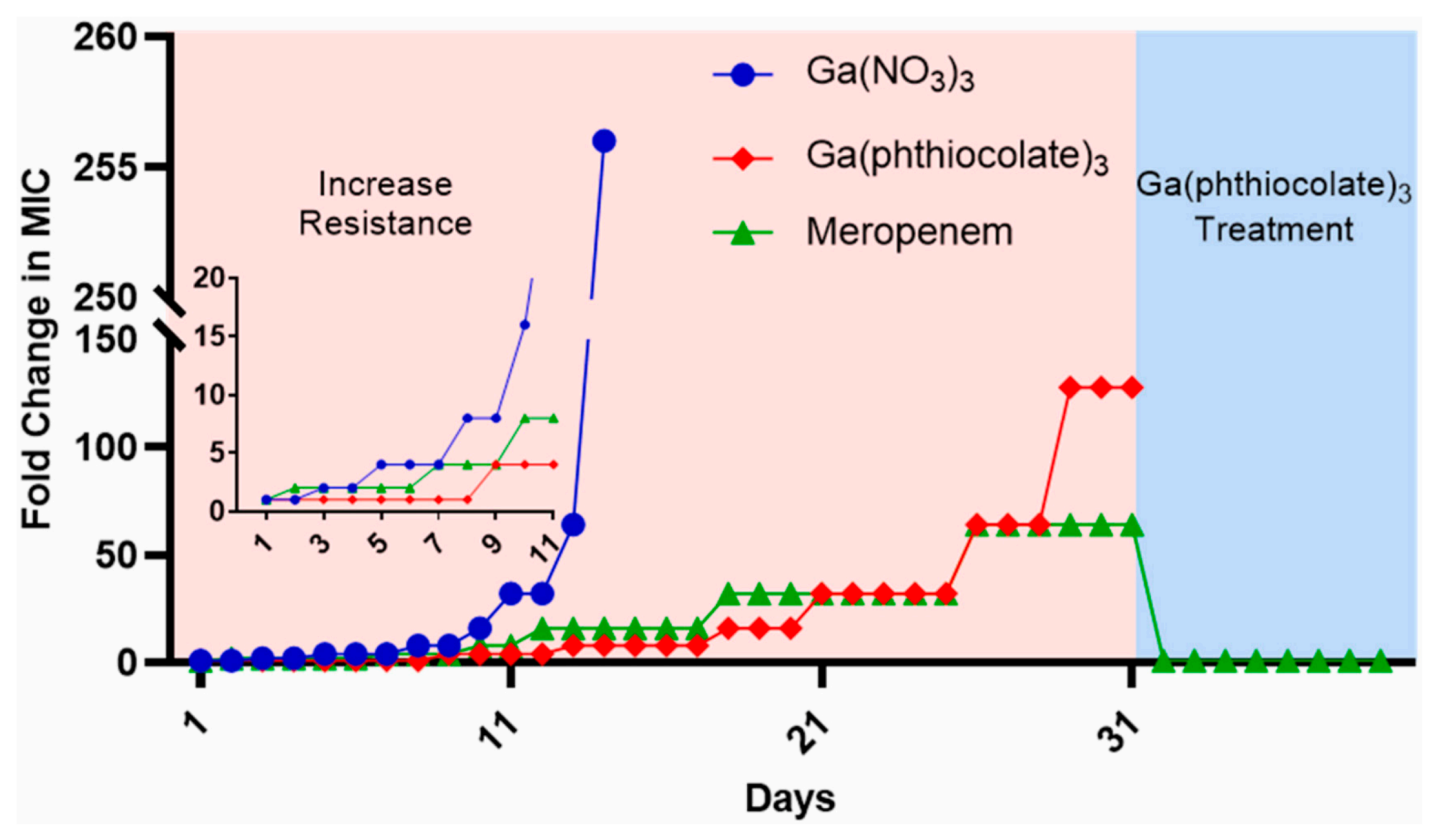
Development of drug resistance in *P. aeruginosa* during prolonged exposure to Ga(phthiocolate)_3_, Ga(NO_3_)_3_, and meropenem. Reproduced from [89], under the Creative Commons Attribution 4.0 International (CC BY 4.0) license.

**Figure 6:**
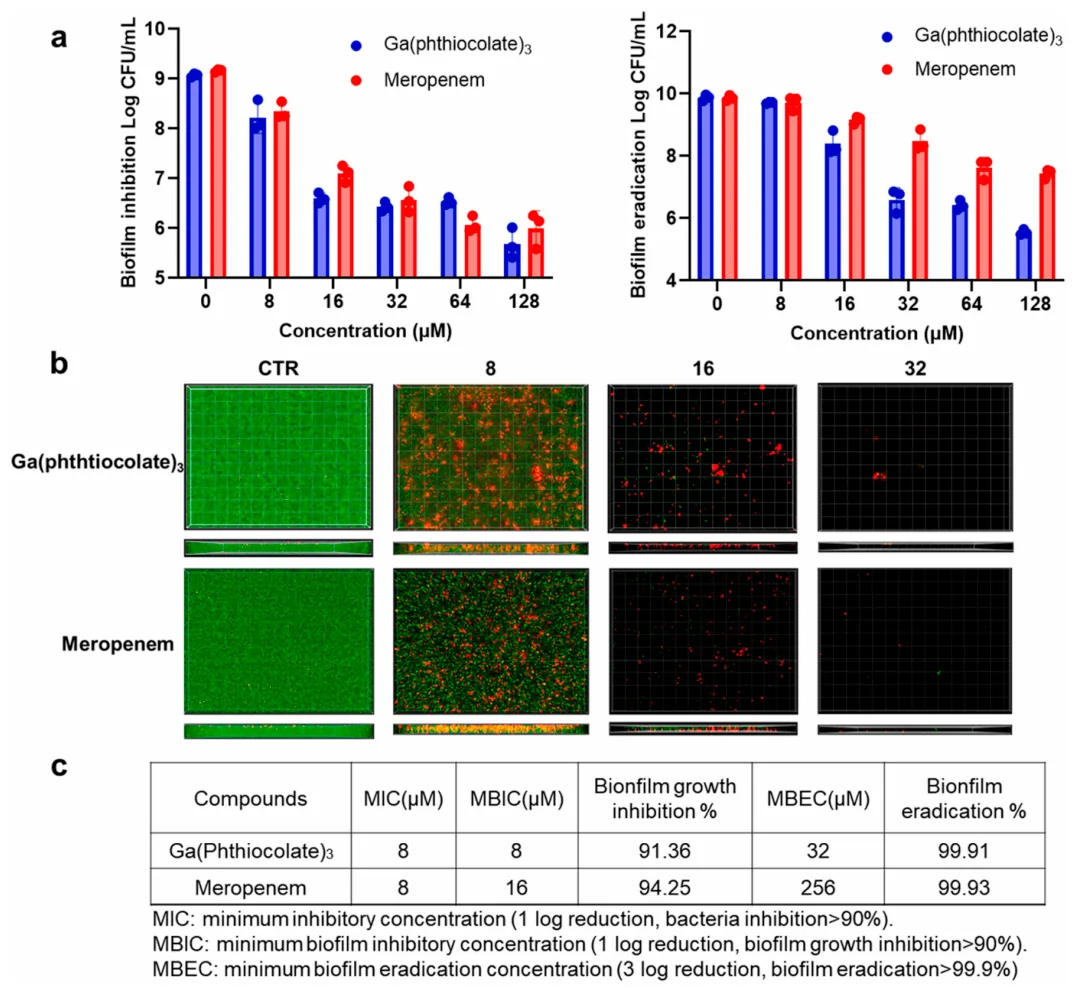
Antibiofilm and biofilm-eradication effects of Ga(phthiocolate)_3_ and meropenem against multidrug-resistant *P. aeruginosa*. (**a**) Quantitative assessment of biofilm inhibition (**left**) and eradication (**right**) in multidrug-resistant *P. aeruginosa* after 24 h of treatment with Ga(phthiocolate)_3_ and meropenem. (**b**) Representative fluorescence microscopy images of multidrug-resistant *P. aeruginosa* biofilms stained with SYTO 9 and propidium iodide. (**c**) Minimum biofilm inhibitory concentration (MBIC) and minimum biofilm eradication concentration (MBEC) values of Ga(phthiocolate)_3_ and meropenem against multidrug-resistant *P. aeruginosa*. Reproduced from [89], under the Creative Commons Attribution 4.0 International (CC BY 4.0) license.

Among the compounds investigated Ga(phthiocolate)_3_ showed particularly strong antimicrobial activity against *P. aeruginosa*. Structure–activity relationship (SAR) analysis suggested that the extended π-conjugated system of phthiocol increases its lipophilicity and improve membrane permeability, contributing to the enhanced antimicrobial activity of its Ga(III) complex. Ga(phthiocolate)_3_ appeared to impose a lower selective pressure for the development of resistance in *P. aeruginosa* and retained its antimicrobial activity against meropenem-resistant strains [89]. This suggests that Ga(phthiocolate)_3_ may act through mechanisms distinct from those of meropenem and may not share the same cross-resistance mechanisms, highlighting its potential against strains resistant to conventional antibiotics (Figure 5). This Ga complex showed strong antimicrobial activity against both planktonic and biofilm-associated *P. aeruginosa*, including a multidrug-resistant strain, with an MIC of 5.0 μg/mL and a minimum biofilm eradication concentration (MBEC) of 20 μg/mL (Figure 6). In comparison, HEK293 human embryonic kidney cells retained >80% viability following 24 h exposure even at 256 μM Ga(phthiocolate)_3_, a concentration approximately 32-fold higher than its antibacterial MIC/MBIC and eightfold higher than its MBEC. Furthermore, Ga(phthiocolate)_3_ showed only limited hemolysis of mouse erythrocytes over the investigated concentration range. Mechanistic studies suggested that its enhanced lipophilicity promoted cellular uptake of Ga(III), allowing greater disruption of iron-dependent bacterial processes and contributing to biofilm eradication. In addition, Ga(phthiocolate)_3_ showed synergistic activity with meropenem, reducing the MIC of meropenem eightfold, while exhibiting low cytotoxicity and minimal haemolytic activity.

Taken together, these findings support the potential of Ga(phthiocolate)_3_ as an antimicrobial agent against drug-resistant bacteria and provide further evidence that rational control of ligand structure and lipophilicity can enhance the antibacterial and antibiofilm activity of Ga(III)-based compounds.

Collectively, these studies demonstrate that the antimicrobial activity of Ga(III) is strongly influenced by the nature of its coordinating ligands, underscoring a shift from the conventional use of gallium salts toward the rational development of ligand-coordinated Ga(III) antimicrobial agents (Table 1). The antimicrobial activity of Ga(III) complexes may depend on their stability and speciation under biological conditions, since ligand dissociation could result in activity arising from the intact complex, released Ga(III), free ligand, or combinations of these species. Therefore, complex stability should be considered when interpreting ligand-dependent antimicrobial mechanisms. Rational ligand selection can influence complex stability, gallium solubility, bacterial uptake, membrane interactions, and targeting of iron-acquisition pathways, while biologically active ligands may introduce complementary antimicrobial mechanisms. The development of siderophore-based and lipophilic Ga(III) complexes, together with the repurposing of pharmacologically established compounds such as KP46, illustrates the versatility of coordination chemistry for optimizing gallium-based antimicrobials. Although lipophilicity can play an important role in the antimicrobial activity of Ga(III) complexes by facilitating interactions with bacterial membranes and potentially enhancing cellular uptake, greater lipophilicity should not be interpreted as necessarily resulting in greater antimicrobial activity. Lipophilicity is commonly quantified using logP or the pH-dependent distribution coefficient (logD). Available data illustrate differences among representative Ga(III) complexes: gallium maltolate has a reported logP of 0.41, KP46 a logP of 0.88, while Ga(phthiocolate)_3_ exhibits an apparent logD_7_._4_ of 2.45. The greater lipophilicity of Ga(phthiocolate)_3_ was associated with enhanced cellular uptake and antimicrobial activity. However, the relationship between ligand structure and antimicrobial efficacy is complex, and increased lipophilicity alone should not be considered a universal predictor of enhanced antimicrobial activity. Greater lipophilicity does not necessarily result in greater antimicrobial potency, nor does lower lipophilicity necessarily correspond to reduced activity, as antimicrobial efficacy is governed by the interplay of multiple physicochemical and biological factors. In addition to lipophilicity, parameters such as complex stability and speciation, aqueous solubility, overall charge, bacterial membrane interactions and cellular uptake, and ligand-derived biological activity may collectively influence antimicrobial performance. Therefore, effective ligand-guided design will require the coordinated optimization of these properties rather than maximizing lipophilicity alone. Establishing systematic structure–activity relationships among these parameters will be essential for developing a more predictive approach to the design of next-generation Ga(III) antimicrobials capable of targeting multidrug-resistant pathogens, disrupting bacterial biofilms, delaying the development of resistance, and potentially enhancing the efficacy of conventional antibiotics. Future systematic studies in which ligand lipophilicity is varied while other structural and coordination parameters are controlled will be important for defining the optimal physicochemical properties of Ga-based antimicrobial agents.

## 7. Conclusions and Future Perspectives

Gallium(III) complexes have gained significant attention as promising antimicrobial agents because Ga(III) can mimic Fe(III), thereby disrupting essential iron-dependent processes within bacterial cells. However, the antimicrobial activity of Ga(III) strongly depends on the properties of its coordinated ligands. Rational ligand design can improve the stability, solubility, cellular uptake, selectivity, and bioavailability of Ga(III) complexes while also facilitating their delivery to bacterial cells.

Different ligand systems, including siderophores, thiosemicarbazones, quinolones, and other biologically active chelators, have demonstrated the potential to enhance the antimicrobial properties of Ga(III). In particular, siderophore-based complexes provide a promising strategy because they can utilize bacterial iron-uptake pathways to increase Ga(III) accumulation within bacterial cells. Furthermore, combining Ga(III) with biologically active ligands or conventional antibiotics may produce multiple antibacterial effects and improve activity against resistant bacteria.

Despite these encouraging findings, several important challenges remain to be addressed. Future studies should focus on understanding the relationship between ligand structure, Ga(III) complex stability, bacterial uptake, and antimicrobial activity. Greater attention should also be given to the stability and speciation of Ga(III) complexes under physiological conditions, their selectivity toward bacterial cells, potential toxicity, and their activity against multidrug-resistant pathogens and biofilms. In addition, more in vivo studies are required to evaluate their pharmacokinetics, biodistribution, safety, and therapeutic efficacy.

Overall, rational ligand design represents a promising strategy for enhancing the antimicrobial activity of Ga(III). Designing ligands that provide strong Ga(III) coordination while enabling selective bacterial targeting and contributing additional antimicrobial functions could facilitate the development of more effective gallium-based therapeutic agents. Further integration of coordination chemistry, microbiology, and drug-delivery approaches will be crucial for advancing promising Ga(III) complexes toward clinically important antimicrobial agents.

## Figures and Tables

**Table 1:** Representative ligand-coordinated Ga(III) complexes investigated as antimicrobial agents, highlighting ligand-design strategies, antimicrobial activity, and proposed biological advantages. MIC values are presented as reported in the original studies and should not be directly compared across compounds because experimental conditions, bacterial strains, growth media, iron availability, and antimicrobial endpoints differ among studies.

Ga(III) Complex	Ligand/Design Strategy	logP	Main Microorganisms Evaluated	Antimicrobial Activity	Key Biological/Design Features	Ref.
Gallium maltolate (GaM)	Maltolate, bidentate O,O-donor ligand forming a neutral Ga(III) complex.	0.41	*Rhodococcus equi*, *Staphylococcus aureus*/MRSA, *Pseudomonas aeruginosa*, *Acinetobacter baumannii*	MIC 8–32 μg/mL against *R. equi*; activity also reported against *S. aureus*/MRSA and *P. aeruginosa*	Coordination with maltolate alters the physicochemical properties of Ga(III) relative to Ga salts and has been associated with improved Ga delivery.	[80,81]
DFO–Ga	Deferoxamine; hydroxamate siderophore-mediated Trojan-horse delivery.	−3.31	*P. aeruginosa*, including clinical isolates	MIC 0.032 mM under low-iron conditions	Exploits bacterial iron-uptake pathways; active against planktonic and biofilm-associated bacteria; enhanced activity with gentamicin.	[82]
Galbofloxacin	Desferrichrome siderophore–ciprofloxacin conjugate incorporating a protease-cleavable linker.	NR	*S. aureus*	MIC_98_ 93 nM, compared with 0.9 μM for ciprofloxacin and 0.93 μM for the apo conjugate.	Siderophore-mediated uptake combines targeted Ga delivery with antibiotic delivery; demonstrated strong in vivo efficacy in a mouse *S. aureus* infection model.	[84]
Pyridine–thiosemicarbazone Ga(III) complexes	N(4)-phenyl-2-formyl-, 2-acetyl-, and 2-benzoylpyridine thiosemicarbazones; N,N,S chelation.	NR	*P. aeruginosa*	Ga(III) coordination significantly increased antibacterial activity relative to Ga(III) and the corresponding free thiosemicarbazones.	Early evidence that coordination chemistry can enhance antimicrobial activity; demonstrates the influence of ligand structure and N,N,S chelation.	[77]
Chalcone–thiosemicarbazone Ga(III) complexes	Pyridyl chalcone-derived thiosemicarbazones with systematic aromatic substitution.	NR	*P. aeruginosa*, *S. aureus*, *Candida albicans*	Ga(III) complexation enhanced antimicrobial activity relative to the corresponding ligand systems.	Provides a ligand-substitution/SAR platform and supports the concept that structural modification of thiosemicarbazones can modulate antimicrobial performance following Ga(III) coordination.	[90]
Pyridoxal–thiosemicarbazone Ga(III) complexes (GaL2, GaL5)	Pyridoxal-derived thiosemicarbazones; ligand-substitution and physiological-speciation-guided design.	NR	Resistant *K. pneumoniae*, *E. coli*, and *P. aeruginosa.*	GaL2/GaL5 MICs 15.63–62.5 μM across resistant Gram-negative strains; Ga(NO_3_)_3_ was >1000 μM under the same conditions.	Strong evidence connecting ligand structure, Ga(III) complex speciation/stability at physiological pH, antimicrobial activity, and selectivity; synergy with meropenem also reported.	[91]
[Ga_2_L_3_(bpy)_2_]	Lawsone-derived pharmacophoric ligand + 2,2′-bipyridine; lipophilic and biologically active ligand system.	NR (insoluble in water but soluble in DMSO)	Drug-resistant *P. aeruginosa* and MRSA	MIC 10 μM (14.8 μg/mL) against *P. aeruginosa* and 100 μM (148 μg/mL) against MRSA	Broad-spectrum activity without requiring iron-limited conditions; proposed dual action involving Ga-mediated disruption of iron metabolism and ligand-associated ROS production; slower resistance development reported.	[87]
KP46 [Ga(8-hq)_3_]	8-Hydroxyquinoline; lipophilic and biologically active chelating ligand; drug-repurposing strategy.	0.88	*A. baumannii*, including carbapenem-resistant *A. baumannii* (CRAB).	Inhibition of planktonic growth and early biofilm development.	8-HQ may contribute intrinsic antimicrobial/metal-chelating activity in addition to coordinating Ga(III); oxidative stress and membrane damage implicated; slower resistance development than meropenem reported.	[88]
Ga(phthiocolate)_3_	Phthiocol; extended π-conjugated lipophilic ligand selected through ligand-structure/lipophilicity-guided design.	2.45	*P. aeruginosa*, including multidrug-resistant strains.	MIC 5.0 μg/mL; MBEC 20 μg/mL against MDR *P. aeruginosa.*	Enhanced lipophilicity was associated with increased cellular uptake; strong antiplanktonic and antibiofilm activity; eightfold reduction in meropenem MIC in combination; low cytotoxicity and minimal hemolysis reported.	[89]

NR—not reported in the literature.

## Data Availability

No new data were created or analyzed in this study. Data sharing is not applicable to this article.

## Abbreviations

The following abbreviations are used in this manuscript:

AMR	Antimicrobial resistance
WHO	World Health Organization
SA	*Staphylococcus aureus*
PA	*Pseudomonas aeruginosa*
MDRSA	Multidrug-resistant *S. aureus*
MRSA	Methicillin-resistant *S. aureus*
VRSA	Vancomycin-resistant *S. aureus*
MDRPA	Multidrug-resistant *P. aeruginosa*
DNA	Deoxyribonucleic acid
FDA	U.S. Food and Drug Administration
MIC	Minimum inhibitory concentration
MBIC	Minimum biofilm inhibitory concentration
MBEC	Minimum biofilm eradication concentration
CRAB	Carbapenem-Resistant *Acinetobacter baumannii*
